# The Novel Small-molecule Annexin-A1 Mimetic, Compound 17b, Elicits Vasoprotective Actions in Streptozotocin-induced Diabetic Mice

**DOI:** 10.3390/ijms21041384

**Published:** 2020-02-18

**Authors:** Sarah A Marshall, Cheng Xue Qin, Maria Jelinic, Kelly O’Sullivan, Minh Deo, Jesse Walsh, Mandy Li, Laura J Parry, Rebecca H. Ritchie, Chen Huei Leo

**Affiliations:** 1 The Ritchie Centre, Department of Obstetrics and Gynaecology, School of Clinical Sciences, Monash University, Clayton, Victoria 3168, Australia; 2 Heart Failure Pharmacology, Baker Heart & Diabetes Institute, Victoria 3004, Australia; 3 Department of Pharmacology, University of Melbourne, Victoria 3010, Australia; 4 Drug Discovery Biology, Monash Institute of Pharmaceutical Sciences, Monash University, Victoria 3052, Australia; 5 Department of Physiology, Anatomy & Microbiology, La Trobe University, Bundoora, Victoria 3086, Australia; 6 School of BioSciences, The University of Melbourne, Victoria 3010, Australia; kelly.osullivan@monash.edu (K.O.);; 7 Science and Math Cluster, Singapore University of Technology & Design, Singapore 487372, Singapore

**Keywords:** diabetes, compound 17b, endothelial dysfunction, FPR1/2

## Abstract

The formyl peptide receptor (FPR) family are a group of G-protein coupled receptors that play an important role in the regulation of inflammatory processes. It is well-established that activation of FPRs can have cardioprotective properties. Recently, more stable small-molecule FPR1/2 agonists have been described, including both Compound 17b (Cmpd17b) and Compound 43 (Cmpd43). Both agonists activate a range of signals downstream of FPR1/2 activation in human-engineered FPR-expressing cells, including ERK1/2 and Akt. Importantly, Cmpd17b (but not Cmpd43) favours bias away from intracellular Ca^2+^ mobilisation in this context, which has been associated with greater cardioprotection in response to Cmpd17b over Cmpd43. However, it is unknown whether these FPR agonists impact vascular physiology and/or elicit vasoprotective effects in the context of diabetes. First, we localized FPR1 and FPR2 receptors predominantly in vascular smooth muscle cells in the aortae of male C57BL/6 mice. We then analysed the vascular effects of Cmpd17b and Cmpd43 on the aorta using wire-myography. Cmpd17b but not Cmpd43 evoked a concentration-dependent relaxation of the mouse aorta. Removal of the endothelium or blockade of endothelium-derived relaxing factors using pharmacological inhibitors had no effect on Cmpd17b-evoked relaxation, demonstrating that its direct vasodilator actions were endothelium-independent. In aortae primed with elevated K^+^ concentration, increasing concentrations of CaCl_2_ evoked concentration-dependent contraction that is abolished by Cmpd17b, suggesting the involvement of the inhibition of Ca^2+^ mobilisation via voltage-gated calcium channels. Treatment with Cmpd17b for eight weeks reversed endothelial dysfunction in STZ-induced diabetic aorta through the upregulation of vasodilator prostanoids. Our data indicate that Cmpd17b is a direct endothelium-independent vasodilator, and a vasoprotective molecule in the context of diabetes.

## 1. Introduction

The formyl peptide receptor (FPR) family belongs to a group of G-protein coupled receptors that play key roles in the regulation and resolution of inflammation. There are three FPRs identified in humans (FPR1, FPR2, and FPR3) [1] all responding to a wide variety of peptide and small-molecule-based agonist and antagonists [2]. FPR1 and FPR2 are expressed in a variety of tissues and cell types, but predominantly in inflammatory cells, such as neutrophils and monocytes [3]. The role of FPR3 is less clear with expression evident on dendritic cells [1,3]. Evidence for an important role of FPRs in promoting anti-inflammatory processes in the cardiovascular system has been well documented. Stimulation of FPR1 protected cardiomyocyte preparations against endotoxic shock, metabolic injury, and ischaemia/reperfusion injury, leading to improved cardiac function [4,5,6]. However, activation of FPR2 caused the phagocytosis of apoptotic neutrophils [7], inhibited neutrophil-platelet aggregation [8], reduced leukocyte adhesion to endothelial cells [9], and attenuated tumour necrosis factor-α-induced oxidative stress and cell adhesion markers in human dermal microvascular endothelial cells [10].

A well-known endogenous anti-inflammatory ligand for FPR is a 37 kDa protein known as annexin-A1 (ANX-A1). It is constitutively expressed in many tissues and cells, including lungs, kidneys, brain, blood vessels, and heart, as well as circulating inflammatory cells [11]. ANX-A1 is an important anti-inflammatory protein in the vasculature as deficiency of ANX-A1 exaggerated inflammatory responses in blood vessels. We have recently demonstrated that ANX-A1 deficiency exaggerates vascular remodelling in the mesenteric artery of diabetic rodents [12]. Annexin-A1 also has significant cardiovascular protective properties, stemming from its ability to resolve inflammation, largely attributed to FPR2 [3]. Recently, small-molecule-based mimetics of FPR1/FPR2 known as Compound 17b (Cmpd17b) [13] and Compound 43 (Cmpd43) were described [14] (Supplementary Figure S1). In human-engineered cells overexpressing FPRs, Cmpd17b and Cmpd43 activate a range of signalling pathways that are relevant for cardioprotection such as pERK1/2, pAkt, cAMP, and intracellular Ca^2+^ mobilization [15]. Interestingly, Cmpd17b, in comparison to Cmpd43, has a 30-fold reduced signalling away from intracellular Ca^2+^ mobilization, a trend that favours cardioprotection. Furthermore, it was demonstrated that Cmpd17b administered during reperfusion *in vivo* reduces cardiac necrosis, inflammation, cardiac remodelling, and improves cardiac function [15]. Endothelial cells release several vasoactive factors that regulate the tone of the underlying smooth muscles cells [16,17,18]. The impact of FPR agonists on the regulation of vascular tone under physiological condition remains unclear and contradicting. For example, several studies reported that the lipid mediator FPR2-selective agonist, lipoxin A4 (LxA4) is co-currently a vasodilator [19,20] and vasoconstrictor [21,22,23]. Given the profound beneficial effects of small-molecule-based FPR agonists in the heart, particularly those exhibiting selectivity away from calcium mobilization, their impact on vascular tone is worthy of investigation.

It is well-established that vascular dysfunction is a critical initiating factor in the development of diabetic-induced cardiovascular diseases [24,25]. Furthermore, hyperglycaemia-induced vascular inflammation and oxidative stress are major contributing factors to the vascular dysfunction in animal models of diabetes [26,27,28,29] and in diabetic patients [30,31]. Specifically, vascular dysfunction is characterized by reduced endothelium-dependent relaxation underpinned by impaired endothelium-derived nitric oxide (NO), prostacyclin (PGI_2_), and/or endothelium-derived hyperpolarization (EDH) in the macro- or microvasculature [32,33,34]. Therefore, activation of FPRs may warrant investigation as a potential novel treatment for diabetes-induced endothelial dysfunction.

Therefore, the main objectives of this study were to: (i) Localise FPR expression in the mouse aorta, (ii) determine if the small-molecule-based FPR-agonists Cmpd17b and Cmpd43 acutely regulate vasculature tone, and (iii) whether or not Cmpd17b and Cmpd43 chronically improve endothelial function of the aorta in a model of type 1 diabetes in male mice.

## 2. Results

### 2.1. Localization of FPR1 and FPR2 in the Aorta

**Figure 1:**
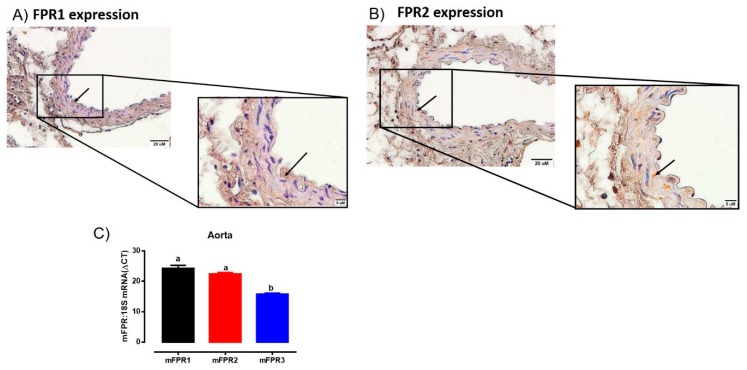
Localisation of FPR1 (**A**) and FPR2 (**B**) protein in the vascular smooth muscle cells in aorta of healthy adult male mice using immunohistochemistry. Arrows indicate positively stained cells. Scale bars = 20 μm, 5 μm. (**C**) Quantitative PCR expression of Fpr1 (*Fpr1*), Fpr2 (*Fpr2*), and Fpr3 (*Fpr3*) mRNA in the aorta. Expression is relative to the housekeeping gene ribosomal 18s. Genes that do not share a letter are significantly different from one another. Values are expressed as mean ± SEM, *n* = 3 per group.

Immunoreactive FPR1 and FPR2 were localized in the aorta of mice (Figure 1). Comparisons between endothelial and vascular smooth muscle cells revealed FPR1 and FPR2 were predominantly localized to the smooth muscle cells, with only very limited immunostaining observed in the endothelial layer (Figure 1A,B). In addition, the intensity of immunostaining for both FPR1 and FPR2 appeared similar in the mouse aorta. Similarly, the mRNA expression of *Fpr1* and *Fpr2* were comparable in the mouse aorta. Interestingly, the gene expression of *Fpr3* was significantly lower than either *Fpr1* or *Fpr2* (Figure 1C). However, due to the absence of a commercially available antibody for FPR3 in mice, it was not possible to assess whether or not FPR3 was also localized to the aorta at present.

### 2.2. Cmpd17b But not Cmpd43 Is a Vasodilator in the Aorta

**Figure 2:**
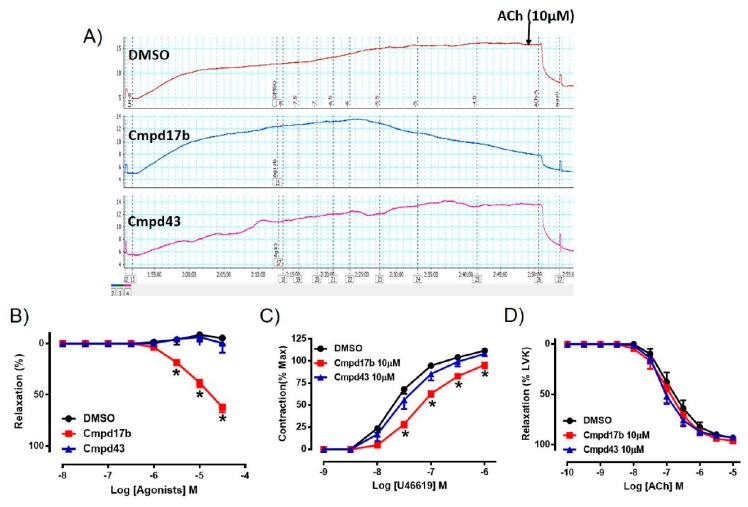
A representative tracing of (**A**) vascular reactivity in the abdominal aorta of male mice during a dose response curve to control (DMSO), Cmpd17b, and Cmpd43, prior to exposure to ACh. Concentration–response curves to (**B**) control, Cmpd17b, and Cmpd43 in the aorta of male mice. Concentration–response curves to (**C**) U46619 and (**D**) ACh from male aorta incubated with DMSO (control), Cmpd17b, or Cmpd43. Values are expressed as mean ± SEM, *n* = 5 per group. Sensitivity (pEC_50_), maximum relaxation (R_max_), and maximum constriction (E_max_) values are shown in Supplementary Table S3. * *P* < 0.05, significantly different to vehicle control.

To assess whether either small molecule compounds could directly induce relaxation, the aorta was preconstricted and then exposed to increasing doses of Cmpd17b, Cmpd43, or vehicle control (<1% DMSO in Krebs). In comparison to control, which maintained precontraction tone, Cmpd17b (but not Cmpd43) produced a concentration-dependent relaxation in the aorta (Figure 2A,B, Supplementary Table S3), suggesting that only Cmpd7b is a direct vasodilator. The sensitivity and maximum relaxation were also significantly different to vehicle control (*P* < 0.0001) and Cmpd43 (Supplementary Table S3). Furthermore, incubation of Cmpd17b (10 µM, the highest concentration studied), but not Cmpd43 at the same concentration (10 µM) acutely significantly shifted the concentration–response curve of U46619 to the right (Figure 2C). Similarly, Cmpd17b significantly decreased the sensitivity (*P* = 0.002) and maximum contraction (*P* = 0.006) to the vasoconstrictor, U46619 (Figure 2C, Supplementary Table S3), but did not affect responses to the endothelium-dependent dilator, ACh (Figure 2D, Supplementary Table S3).

### 2.3. Mechanisms of Cmpd17b-Induced Relaxation

**Figure 3:**
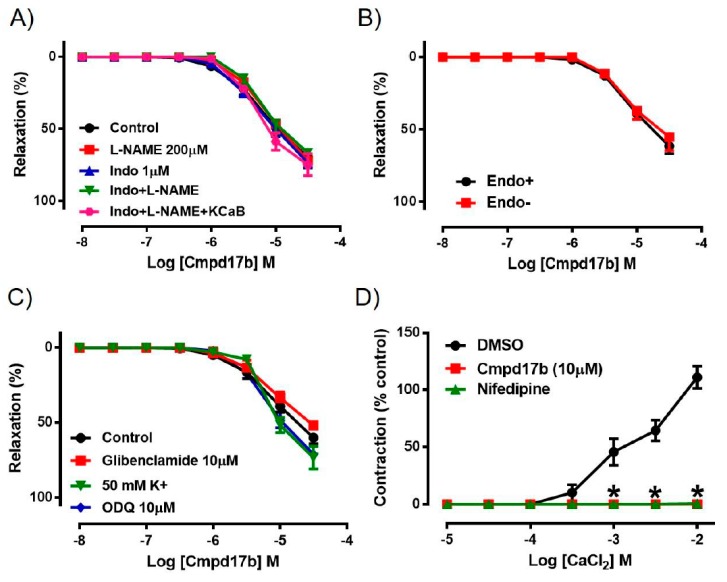
Concentration–response curves to (**A**) Cmpd17b in the presence of DMSO (control), L-NAME (NOS inhibitor), Indo (non-selective COX inhibitor), L-NAME+Indo, or LNAME+Indo+KCaB (inhibitors of K_Ca_3.1 and K_Ca_2.x channels) in the aorta of male mice. Concentration–response curves to Cmpd17b in (**B**) endothelium intact and endothelium denuded aorta or (**C**) in the presence of 50mM K^+^, ODQ (soluble guanylate cyclase inhibitor) or glibenclamide (ATP-sensitive potassium channel blocker). Concentration–response curves to (**D**) CaCl_2_ in aorta treated with control (DMSO), Cmpd17b, or nifedipine (calcium channel blocker). * *P* < 0.05, significantly different to control. Values are expressed as mean ± SEM, *n* = 3–12 per group. Sensitivity (pEC_50_), maximum relaxation (R_max_), and maximum constriction (E_max_) values are shown in Table 1.

To explore the potential involvement of endothelium-derived factors in mediating Cmpd17b-evoked vasorelaxation, responses to Cmpd17b were evaluated in the presence of pharmacological blockers such as L-NAME, Indo, Indo+L-NAME, or Indo+L-NAME+KCaB. However, neither the presence of these inhibitors alone nor combination of these inhibitors had any effect on Cmpd17b-induced relaxation, indicating that endothelium-derived relaxing factors do not contribute to the vasodilator effects of Cmpd17b (Figure 3A, Table 1). Similarly, responses to Cmpd17b were comparable between endothelium-intact and endothelium-denuded aortic rings, further implying its mechanism(s) of action are not dependent on the endothelium (Figure 3B, Table 1). These observations that the vasodilator responses were independent of the endothelium are consistent with (and perhaps a consequence of) the low level of constitutive FPR expression on endothelial cells relative to the vascular smooth muscle.

Incubation with elevated K^+^ concentration (50 mmol·L^−1^), ODQ, or glibenclamide also had no significant effect on the responses to Cmpd17b in the mouse aorta (Figure 3C, Table 1). However, when exposed to increasing concentrations of CaCl_2_ in aorta primed with elevated K^+^ concentration (100 mmol·L^−1^), the presence of Cmpd17b or the calcium channel blocker nifedipine abolished Ca^2+^-induced contraction when compared to DMSO control (Figure 3D, Table 1).

### 2.4. Systemic Characteristics of STZ-Induced Diabetes

**Figure 4:**
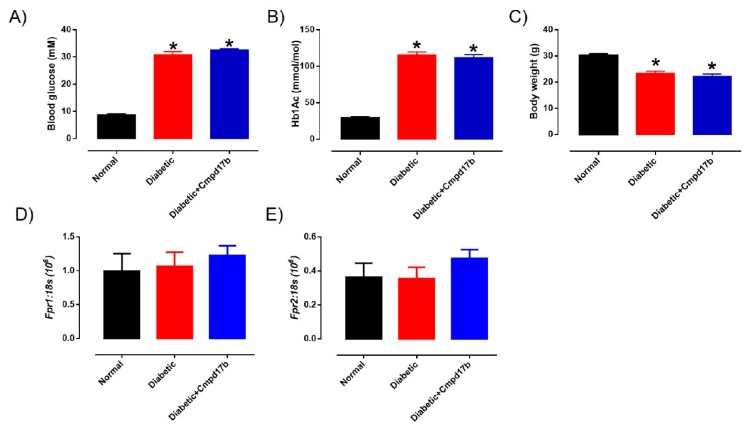
A comparison of (**A**) blood glucose (mM), (**B**) glycated haemoglobin level (HbA_1c_; mM), and (**C**) body weight (g) from citrate buffer (normal) or streptozotocin (diabetic)-treated male mice with or without Cmpd17b. Quantitative PCR expression of (**D**) *Fpr1* and (**E**) *Fpr2* mRNA in the aorta of citrate buffer (normal) or streptozotocin (diabetic)-treated male mice with or without Cmpd17b. Values are expressed as mean ± SEM, *n* = 8–12 per group. * *P* < 0.05 significantly different from values in normal mice.

The blood glucose, glycated haemoglobin (HbA_1c_) levels, and body weight of the mice are shown in Figure 4. Sixteen weeks after treatment with STZ, the blood glucose and glycated haemoglobin of diabetic mice were significantly (*P* < 0.0001) greater than control mice (Figure 4A,B). Similarly, body weights in diabetic mice were significantly lower than control mice (Figure 4C). Cmpd17b treatment *in vivo* for eight weeks had no significant effects on any of these parameters (Figure 4A–C).

### 2.5. Investigating Mechanisms of Cmpd17b Action in Diabetes

**Figure 5:**
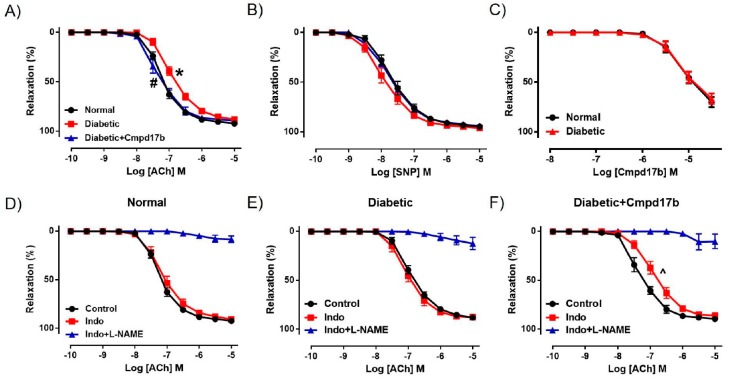
Concentration–response curves to (**A**) ACh, (**B**) SNP, and (**C**) Cmpd17b in the aorta of citrate buffer (normal) or streptozotocin (diabetic)-treated male mice with or without Cmpd17b. Concentration–response curves and to ACh in the presence or absence of Indo (non-selective COX inhibitor) or Indo+L-NAME (NOS inhibitor) in the aorta of citrate buffer (normal) (**D**), streptozotocin (diabetic) (**E**), or streptozotocin (diabetic) and Cmpd17b (**F**) treated male mice. Values are expressed as mean ± SEM, *n* = 4–8 per group. Sensitivity (pEC_50_) and maximum relaxation (R_max_) values are shown in Table 2. *P* < 0.05 significantly different from pEC_50_ values in (*) normal mice or (#) diabetic mice. ^ *P* < 0.05 significantly different from pEC_50_ values in control within the same treatment group.

We further investigated the mechanisms underpinning the reversal of endothelial dysfunction after eight weeks of Cmpd17b treatment using pharmacological inhibitors. In the presence of the COX inhibitor Indo, ACh-mediated relaxation was not affected in the aorta of control mice (Figure 5D, Table 2), suggesting that the contribution of COX to endothelium-dependent relaxation has little to no role in the aorta. Responses to ACh were almost completely abolished by the presence of Indo+L-NAME (Figure 5D, Table 2), implying that endothelium-dependent relaxation in the aorta is almost completely controlled by NO, with minimal contribution from prostanoids or EDH. Despite a reduction in ACh-evoked relaxation in the diabetic aorta, neither the sensitivity nor maximum relaxation to ACh (Figure 5E,F, Table 2) were affected after incubation with Indo. Similarly, responses to ACh were almost completely abolished in the presence of Indo+L-NAME (Figure 5E,F, Table 2). Interestingly, the aorta from Cmpd17b-treated diabetic mice had a reduced sensitivity (*P* = 0.017) but not maximum relaxation to ACh after incubation with Indo (Figure 5F, Table 2), indicating that Cmpd17b treatment increases the contribution of prostanoids to endothelium-dependent relaxation in the diabetic aorta. The remaining responses to ACh were almost completely abolished by the presence of Indo+L-NAME (Figure 5F, Table 2).

**Figure 6:**
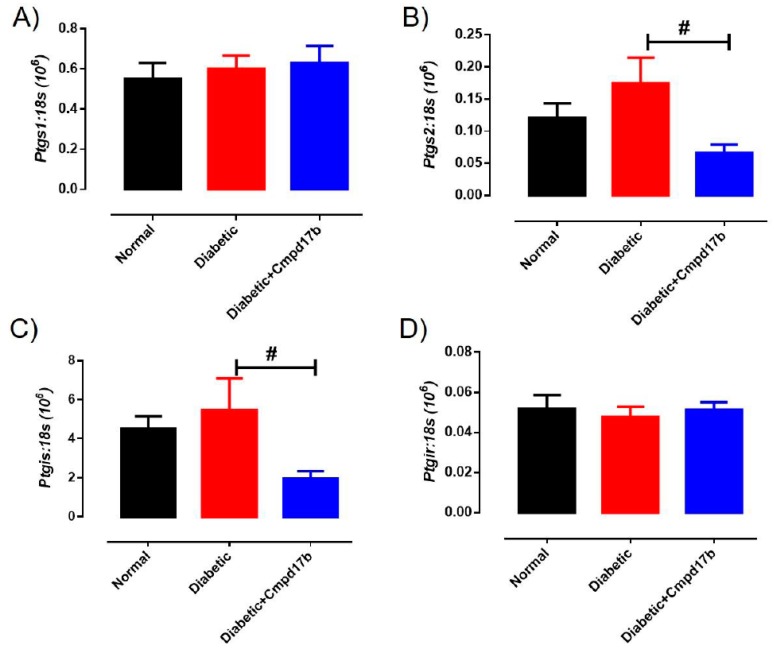
Quantitative PCR expression of (**A**) *Ptgs1*, (**B**) *Ptgs2*, (**C**) *Ptgis,* and (**D**) *Ptgir* mRNA in the aorta of citrate buffer (normal), or streptozotocin (diabetic)-treated male mice with or without Cmpd17b. Values are expressed as mean ± SEM, *n* = 6–12 per group. # *P* < 0.05 significantly different between diabetic mice and diabetic mice treated with Cmpd17b.

**Figure 7:**
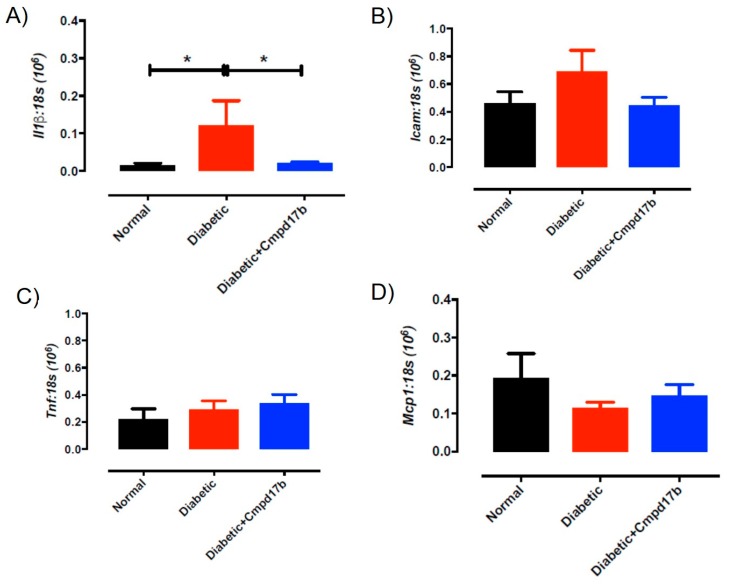
Quantitative PCR expression of (**A**) *Il1β*, (**B**) *Icam*, (**C**) *Tnf,* and (**D**) *Mcp1* mRNA in the aorta of citrate buffer (normal), or streptozotocin (diabetic)-treated male mice with or without Cmpd17b. Values are expressed as mean ± SEM, *n* = 6–12 per group. * *P* < 0.05 significantly different to diabetic mice.

Further investigations into prostanoid synthesis-related gene expression in the aorta revealed that diabetes and Cmpd17b treatment had no effect on *Cox1* (Figure 6A), *Cox2* (Figure 6B), or *Ptgis* (Figure 6C) compared to aorta from control mice. Cmpd17b treatment also had no effect on *Cox1* expression (Figure 6A) but significantly (*P* = 0.008) reduced *Cox2* (Figure 6B) and *Ptgis* expression (*P* = 0.029; Figure 6C) in the aorta of diabetic mice. Lastly, the expression of the prostacyclin receptor, *Ptgir,* was not affected by either diabetes or Cmpd17b treatment (Figure 6D). Interestingly, the gene expression of the pro-inflammatory cytokine, IL1β, was significantly (*P* < 0.05) increased in the diabetic mouse aorta compared to control mice. This upregulation of IL1β expression was abrogated with Cmpd17b treatment in the diabetic mouse (Figure 7A). Neither the expression of the ICAM-1, TNFα, nor MCP-1 was affected by either diabetes or Cmpd17b treatment (Figure 7B–D).

## 3. Discussion

**Figure 8:**
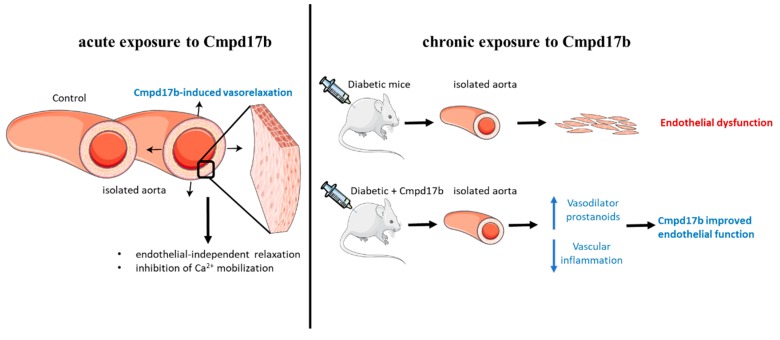
Summary of the impact of FPR agonist Cmpd17b on acute and chronic exposure in isolated mouse aorta. Cmpd17b induced endothelial independent relaxation acutely, and improved endothelial function in diabetic aorta chronically.

This study aimed to establish whether small molecule agonists of FPR1/FPR2 have beneficial vascular effects in the mouse aorta. In this study, FPR1 and FPR2 were primarily localised to the smooth muscle cells in the aorta but only Cmpd17b (and not Cmpd43) was able to induce endothelium-independent relaxation in the mouse aorta (as shown in Figure 8). This is likely, in part, underpinned by the inhibition of Ca^2+^ mobilisation via voltage-gated calcium channels. In addition to being a direct vasodilator via actions on the vascular smooth muscle, Cmpd17b is also a vasoprotective molecule in the setting of diabetes. Specifically, Cmpd17b treatment for eight weeks *in vivo* reversed diabetes-induced endothelial dysfunction by increasing the contribution of vasodilator prostanoids to endothelium-dependent relaxation in the mouse aorta. Furthermore, the vasodilator capacity of Cmpd17b was also maintained and not reduced in the diabetic aorta (as shown in Figure 8).

FPRs are known to be a promiscuous receptor family [1,2,3], which can be activated by either lipid or protein ligands to trigger distinct downstream pro- or anti-inflammatory responses (specific to each particular ligand). Previous studies have reported inconsistent and contradictory findings regarding the vascular function of FPR agonists. Specifically, the lipid mediator FPR2-selective agonist, lipoxin A4 (LxA4), induces endothelium-dependent relaxation in preconstricted rat aorta, mesenteric arteries, and human omental veins but not arteries [19,20]. On the contrary, other studies demonstrated that LxA4 selectively activates FPR2 to induce concentration-dependent contractions via reactive oxygen species production and RhoA/Rho kinase-dependent pathway in the rat aorta [21,22,23]. Similarly, intravenous administration of LxA4 *in vivo* dose-dependently constricted mesenteric arterial bed [35]. In this study, we utilised two small-molecule FPR agonists, Cmpd17b and Cmpd43. Cmpd17b has been previously shown to exhibit a biased signalling profile in engineered Chinese hamster ovary cells overexpressing human FPR1 or FPR2 and cardiomyocytes [15], a property not shared by Cmpd43. The impact of either agonist in blood vessels were, however, not previously known. Specifically, we demonstrated that only Cmpd17b causes concentration-dependent relaxation in the mouse aorta, and antagonised U46619-evoked contraction, indicating that Cmpd17b is a direct vasodilator. Furthermore, acute incubation of Cmpd17b had no effect on ACh-evoked endothelium-dependent relaxation. In contrast, acute incubation of LxA4 potentiated phenylephrine-induced contraction and caused endothelial dysfunction in the rat aorta [22]. Taken together, activation of FPRs by different types of ligands appears to induce distinct effects in the blood vessels.

Earlier studies demonstrated that activation of FPRs by LxA4 causes endothelium-dependent relaxation, which is primarily underpinned by NO in the rat aorta [20] and EDH in the human omental veins [19]. In this study, we demonstrated that vascular relaxation to Cmpd17b was still maintained after inhibition of NO and the sGC signalling pathway, indicating that Cmpd17b is not dependent on the NO pathway. Similar results were obtained after blockade of COX enzymes, and combination of small and intermediate conductance calcium-activated potassium channels, suggesting that there is no involvement of COX-derived prostanoids or endothelium-dependent hyperpolarisation. Consistent with the inhibitors data, endothelial removal also had no effect on Cmpd17b induced relaxation, confirming for the first time that Cmpd17b acts on vascular smooth muscle to cause relaxation in the mouse aorta.

Given that potassium channels are widely expressed in the vascular smooth muscle cells and have an important contribution to vascular tone, we explored whether Cmpd17b-mediated relaxation involved the opening of potassium channels, thereby causing hyperpolarisation and vasorelaxation [36,37]. In the presence of high potassium depolarising solution or the ATP-sensitive potassium channel inhibitor, glibenclamide, Cmpd17b-induced relaxation remained unaffected. Collectively, these results highlight that there is little to no involvement of potassium channels to Cmpd17b-mediated relaxation in the mouse aorta. The annexin family are Ca^2+^ binding proteins [38] and Cmpd17b exhibits a biased signalling response away from Ca^2+^ as previously mentioned [15]. This suggests that Cmpd17b vascular response may also be mediated through the regulation by Ca^2+^ handling or mobilisation. In blood vessels, voltage-gated calcium channels are critical to the maintenance of vascular tone; whereby the depolarisation of the vascular smooth muscle cell membrane leads to opening of voltage-gated calcium channels and a subsequent rise in intracellular Ca^2+^ concentration, resulting in contraction. Conversely, hyperpolarization causes closure of voltage-gated calcium channels, resulting in a reduction of intracellular Ca^2+^ and vasorelaxation [37,39]. In the present study, the aorta was incubated in Ca^2+^-free Krebs and depolarized with K^+^ to open voltage-gated calcium channels, and then a contractile response was initiated by the addition of extracellular Ca^2+^. This was done in the absence and presence of Cpmd17b. Indeed, Cmpd17b incubation completely blocked Ca^2+^-induced contraction of the aorta. This effect was comparable to the positive control, nifedipine (voltage-gated calcium channel blocker). Taken together, these results suggest that Cmpd17b may be involved in the inhibition of Ca^2+^ mobilisation via voltage-gated calcium channels in the smooth muscle cells of the aorta. However, its ability to mediate Ca^2+^ mobilization was not assessed in vascular cells and was beyond the scope of this study.

Earlier studies have reported that activation of FPR results in anti-inflammatory responses in endothelial cells [10,40]. In the present study, diabetes caused the upregulation of the expression of the pro-inflammatory cytokine, IL1β, contributing to a selective impairment of endothelium-dependent relaxation, indicating endothelial dysfunction as previously described [41,42,43,44,45,46]. Chronic treatment with Cmpd17b for eight weeks reduced the IL1β expression and reversed endothelial dysfunction, suggesting an anti-inflammatory effect of Cmpd17b that is independent of any changes to metabolic profile [47]. Although Cmpd17b treatment reverses endothelial dysfunction, the underlying mechanism of this protection remains unknown. The mechanisms underpinning diabetes-induced endothelial dysfunction is multifactorial and may be attributed to the impairment of NO, PGI_2_, and EDH [27,28,42,43,46]. In this study, endothelial dysfunction in the diabetic aorta was primarily underpinned by impairment of NO-mediated relaxation. This finding is consistent many earlier reports that diabetes impaired NO bioactivity through increased oxidative stress and endothelial NOS uncoupling [27,28,42,43,46,48]. Interestingly, in Cmpd17b-treated diabetic mice, there is an increased contribution of a prostanoid component that is usually absence in normal or diabetic mice, suggesting that Cmpd17b treatment improves endothelial function in diabetes by upregulating the contribution of vasodilator prostanoids, likely to be PGI_2_.

To further explore the role of prostanoids in the aorta, we assessed expression of prostanoid associated genes. Cyclooxygenases are the first enzymes involved in the biosynthetic pathway that lead to prostanoid formation from arachidonic acid. There are two isoforms of COX, the constitutive isoform *Ptgs1* is primarily involved in vascular homeostasis while the inducible isoform, *Ptgs2*, is often induced by inflammatory stimuli [49]. Neither of these prostanoid-associated genes were affected by diabetes. This is consistent with our functional data, where there is little to no contribution of prostanoid to endothelium-dependent relaxation in the normal and diabetic aorta. After Cmp17b treatment, the *Ptgs2* expression is downregulated in the aorta, possibly underpinned by the anti-inflammatory effects of FPR agonists. Specifically, this effect of Cmpd17b may be independent of diabetes as it has been reported that upregulation of the endogenous FPR agonist, Annexin A1, reduces *Ptgs2* expression in lung cancer cells [50]. Despite observing a greater functional contribution of prostanoids to endothelium-dependent relaxation in Cmpd17b-treated diabetic mice, a compensatory downregulation of mRNA for prostacyclin synthase (PTGIS) was observed, without affecting *Ptgs1* expression. As observed in earlier studies, increased production of prostanoid metabolites is often accompanied by downregulation of prostanoid synthesizing enzymes such as *Ptgis* [51]. Therefore, it is logical to hypothesise that Cmpd17b treatment may be increasing the production of vasodilator prostanoids, likely via *Ptgs1* to enhance endothelium-dependent relaxation in diabetes, which results in the compensatory downregulation of *Ptgis* gene expression. The limitation of this study is that we are not able to perform additional experiments to directly measure PGI_2_ production or protein expression/activity of the prostanoid enzymes due to the limited tissue availability.

## 4. Materials and Methods

### 4.1. Animals

The first arm of this study used healthy male C57/Bl6J mice aged 3-5 months, which were housed in the Animal House Facilities of University of Melbourne in a 12 h light and dark cycle at 20 °C, with standard food pellets (Barastock, VIC, Australia) and water provided ad libitum. All *ex vivo* animal experiments were performed after the mice were anesthetized under 2% isoflurane (Univentor 400, Agnthos, AB, Sweden) in oxygen via inhalation followed by cervical dislocation. Male mice vascular tissues were scavenged from excessive animals on an existing ethics approved by The Faculty of Science, University of Melbourne Animal Experimental Ethics Committee (AEEC #0911478.1 or #1212387.3). All experiments were conducted in accordance with the Australian Code of Practice and the National Health and Medical Research Council, ARRIVE guidelines and Directive 2010/63/EU of the European Parliament in the protection of animal used for scientific purpose.

### 4.2. Isolation of Aorta

The whole aorta (thoracic and abdominal) was isolated and immediately placed in ice cold Krebs (physiological saline solution: PSS (mmol/L^−1^): 120 NaCl, 5 KCl, 1.2 MgSO_4_, 1.2 KH_2_PO_4_, 25 NaHCO_3_, 11.1 D-glucose, 2.5 CaCl_2_). The aorta was carefully cleared of fat and connective tissue under a dissection microscope. The abdominal aorta was cut into 2mm rings and used for functional studies, while the remaining thoracic aorta was snap frozen in liquid nitrogen for later gene analysis or fixed in neutral-buffered formalin, embedded in paraffin (Alfred Pathology Service, Melbourne, VIC, Australia), and then sectioned at 4 µm.

### 4.3. Immunohistochemistry

Mouse aorta sections were deparaffinised, rinsed, and antigen retrieval was performed by incubating the slides for 20 min at 95 °C in 0.01% citrate buffer (CB). Sections were blocked in 5% normal goat serum (NGS) in 0.01% phosphate buffered saline (PBS)/Tween 20 for 1 h. To detect the expression of FPR1 and FPR2 in the vessel, sections were then incubated overnight at 4 °C with either FPR1 (1:100 in 5% NGS and 0.01% PBS-T, #orb13410, Biorbyt, Cambridge, UK) or FPR2 primary antibody (1:100 in 5% NGS and 0.01% PBS-T, BS-3654R-100UL, Bioss, Woburn, MA, USA). Representative images were photographed as described previously [52,53].

### 4.4. Quantitative Real-Time PCR

Frozen blood vessels from 3 mice were pooled (considered as *n* = 1) and placed in pre-chilled Wig-L-Bug^®^ capsules with a silver ball bearing and pulverized in a Digital Wig-L-Bug^®^ amalgamator (Dentsply-Rinn, Elgin, IL, USA). Pulverized tissues were resuspended in 1 ml TriReagent (Ambion Inc., Scoresby, VIC, Australia) and total RNA was then extracted as described previously [54,55,56,57]. RNA pellets were resuspended in 11 µL RNA Secure™ (Ambion). Quality and quantity of RNA was analysed using the NanoDrop ND1000 Spectrophotometer (Thermo Fischer Scientific Australia Pty Ltd., Scoresby, VIC, Australia) with A260:A280 ratios > 1.8 indicating sufficient quality for qPCR analysis. First-strand cDNA synthesis used 1 µg of total RNA in a 20 µL reaction containing random hexamers (50 ng/µL) and 200 units of Superscript™ III (Invitrogen, Mulgrave, VIC, Australia). Mouse-specific forward/reverse primers (GeneWorks, Thebarton, SA, Australia) were generated from GenBank (Supplementary Table S1). Real-time PCR reaction was determined by SYBR green chemistry using the Applied Biosystems 7500 fast real-time PCR system with triplicate samples of 12.5 µL containing SYBR Green PCR Master Mix (Applied Biosystems, Scoresby, VIC, Australia) and 10 µM (gene of interest) or 1 μmol/L^−1^ (18S) of primers. Expression of *Fpr1*, *Fpr2,* and *Fpr3* was assessed by qPCR using 2^−∆∆*C*t^ quantification cycle (C_t_) method with ribosomal 18S (Rn18s) as the endogenous reference gene. For each sample, the mean *Rn18s* C_q_ triplicate value was subtracted from the mean gene of interest triplicate C_t_ value and to normalize the gene of interest expression to the reference gene. These normalised data (∆C_t_) were then analysed using the 2^−∆∆*C*t^ method and presented as mean ± SEM. Negative template controls substituting cDNA with water or reverse transcriptase negative controls substituting the reverse transcriptase in the cDNA synthesis were included on each plate. For the amplification of *Rn18s,* cDNA was diluted 1:10 in pure water, while all other genes were amplified in neat cDNA.

### 4.5. Assessment of Vascular Reactivity

The aortae were mounted on a wire myograph (model 610M; Danish Myo Technology, Aarhus, Denmark) as described previously [41,58,59,60] with the following modifications. Briefly, the aorta was allowed to stabilize at zero tension for 15 min, followed by a 30 min equilibrium at 5mN. Myography experiments were performed at 37 °C while bubbled continuously with carbogen (95% O_2_ and 5% CO_2_). Changes in isotonic tension were recorded using Powerlab/LabChart data acquisition system (AD Instruments, Bella Vista, NSW, Australia).

After equilibration, aortic rings were maximally contracted (E_max_) with the thromboxane A2 mimetic, U46619 (1 mol/L^−1^). After plateauing, the aorta was washed with Krebs. After submaximally preconstricting arteries with U46619 to approximately 50%–70% of E_max_, the endothelium-dependent relaxant acetylcholine (ACh; 10 μmol/L^−1^) was applied to induce relaxation. Arteries with >90% relaxation were deemed suitable for further analysis. The level of precontraction used when examining vasodilator function was kept constant, with U46619 used for preconstriction.

To assess vasodilator effects of Cmpd17b and Cmpd43, blood vessels were pre-contracted to a similar level (50%–70% of E_max_) using U46619 (0.01–0.3 μmol/L^−1^), followed by cumulative concentration–response curves to vehicle control (dimethyl sulfoxide; DMSO), Cmpd17b (10 nmol/L^−1^–30 μmol/L^−1^), or Cmpd43 (10 nmol/L^−1^–30 μmol/L^−1^). In addition, responses to U46619 or ACh were examined after 20 mins incubation with vehicle control (DMSO), Cmpd17b (10 μmol/L^−1^) or Cmpd43 (10 μmol/L^−1^). In addition, responses to Cmpd17b were examined after 20 mins incubation with different combinations of pharmacological blockers including the nitric oxide synthase (NOS) inhibitor, L-NAME (200 μmol/L^−1^), the cyclooxygenase (COX) inhibitor, indomethacin (Indo, 1 μmol/L^−1^), a combination of both, or a combination with the small conductance calcium-activated potassium channel (SK_Ca_) inhibitor apamin (1 μmol/L^−1^) and the intermediate conductance calcium-activated potassium channel (IK_Ca_) inhibitor, TRAM34 (1 μmol/L^−1^) labelled as KCaB. Further work involved investigating responses to Cmpd17b after 20 mins incubation with global K^+^ channel blockade (50 mmol/L^−1^), the soluble guanylate cyclase inhibitor, 1H-[1,2,4]oxadiazolo [4,3-a]quinoxalin-1-one (ODQ, 10 μmol/L^−1^), or the ATP-sensitive potassium (K_ATP_) channel blocker, glibenclamide (10 μmol/L^−1^). In a separate set of experiments, arteries with or without the endothelium (denuded) were also exposed to Cmpd17b to establish whether Cmpd17b was dependent on the endothelium. Arteries with less than 10% relaxation to ACh were considered to be endothelium denuded.

To further explore voltage gated Ca^2+^ channel involvement, aortae were incubated in Ca^2+^-free EGTA (2mM)-containing Krebs PSS for 40 minutes. This buffer was replaced with Ca^2+^-free potassium containing (100 mmol/L, isoosmotic replacement of Na^+^ with K^+^) PSS and cumulative concentration–response curves to calcium chloride (CaCl_2_; 10 μmol/L to 10mmol/L) performed after 20 min incubation in DMSO, Cmpd17b (10 μmol/L), or the calcium channel blocker nifedipine (10 μmol/L).

### 4.6. Induction of Type 1 Diabetic Mice

All procedures of the second arm of this study were performed in accordance with the Alfred Medical Research and Education Precinct (AMREP) animal ethics regulations and approved by the AMREP Animal Ethics Committee (E/1535/2015/B), ARRIVE guidelines and Directive 2010/63/EU of the European Parliament in the protection of animal used for scientific purpose. All mice were housed in the AMREP animal facility and all experiments conducted in accordance with the Australian Code of Practice and the National Health and Medical Research Council. Five to six mice were housed per cage and maintained under a 12-hour-light-dark cycle. Normal chow diet and water were provided ad libitum.

Six-week-old C57/Bl6 mice were randomly allocated to either the diabetic or non-diabetic group. Following overnight starvation, mice were administered either streptozotocin (STZ; 55mg/kg body weight in 0.1mol/L citrate vehicle, pH 4.5; Sigma- Aldrich, St Louis, MI, USA) or vehicle control (CB) via intraperitoneal (i.p.) injection for five consecutive days [61]. Non-diabetic mice were injected with CB. Two weeks following the induction of diabetes, blood was collected from the saphenous vein on a fortnightly basis, and blood glucose concentrations determined using a handheld Accu-check^®^ Performa glucometer (Roche Diagnostics, Sandhofer, Mannheim, Germany). Mice with readings >24 mmol/L were considered diabetic. Body weight was also recorded. The FPR agonist Cmpd17b was synthesised by Anthem Biosciences (Anthem Biosciences Bengaluru, Karnataka, Bangalore, India) and was dissolved in 10% DMSO solvent (AMRESCO^®^, Solon, OH, USA) and sonicated to yield a suspension. Cmpd17b was added to 0.8% PBS/Tween (Sigma-Aldrich, St Louis, MI, USA) and administered at a dose of 50mg/kg/day for a total of eight weeks via i.p. injection. An equivalent volume of DMSO/PBS Tween vehicle was administered to diabetic mice allocated to the vehicle treatment group. This dose and concentration was previously used in the model of myocardial infarction with no adverse effects reported [15].

### 4.7. Tissue Collection from Diabetic and Non-Diabetic Mice for Vascular Analysis

Following i.p. injection of Ketamine/Xylazine (KX cocktail; 60/6 mg/kg Lyppard, Keysborough, Victoria, Australia), cardiac puncture was performed to collect whole blood using a heparinised 1 mL syringe (5000 IU, Pfizer, West Ryde, New South Wales, Australia). Blood glucose was measured as described above, and a 2 μL aliquot of blood was used for HbA_1c_ measurement using cobas-b-101-point of care system (Roche Holdings AG, Basel, Switzerland). The aorta was collected and prepared as described above.

### 4.8. Vascular Reactivity of Aorta from Diabetic Mice

Vascular reactivity was assessed in the abdominal aorta of CB-, STZ-, and STZ+Cmpd17b-treated mice [41,42,43]. The aortae were pre-contracted to 50%–70% of E_max_ using U46619 (0.01 μmol/L^−1^–0.3 μmol/L^−1^), followed by cumulative concentration–response curves to ACh (0.1 nmol/L^−1^–10 mol/L^−1^) and the endothelium-independent relaxant, sodium nitroprusside, (SNP; 0.1 nmol/L^−1^–10 mol/L^−1^) to test endothelial and smooth muscle function, respectively. In a separate set of experiments, to confirm the vasodilator effects of Cmpd17b on the aorta of diabetic mice, aortae were pre-contracted using U46619 (0.01 μmol/L^−1^–0.3 μmol/L^−1^), followed by cumulative concentration–response curves to Cmpd17b (10 nmol/L^−1^–30 μmol/L^−1^) in control and STZ-treated mice. Then, aortic rings from all three treatment groups were incubated for 20 minutes in either Indo (1 mol/L^−1^) or a combination of Indo with L-NAME (200 μmol/L^−1^), prior to exposure to ACh (0.1 nmol/L^−1^–10 mol/L^−1^).

### 4.9. Quantitative PCR from Diabetic Mice

RNA extraction and cDNA synthesis were performed as described above, except mouse aorta were not pooled. Each mouse represented an *n* = 1. Then, qPCR was performed to assess the relative expression of formyl peptide receptor 1 (*Fpr1*), formyl peptide receptor 2 (*Fpr2*), cyclooxygenase-1 (*Cox-1*), cyclooxygenase-2 (*Cox-2*), prostaglandin I2 receptor (*Ptgir*), prostacyclin synthase (*Ptgis*), tumour necrosis factor alpha (*tnfa),* intercellular adhesion molecule-1 (*icam-1),* monocyte chemoattractant protein-1 (*mcp-1*), and interleukin-1β (*IL-1β*) in the thoracic aorta of mice treated with CB, STZ, or STZ+Cmpd17b. Due to limited tissue availability, the number of genes investigated was restricted. Forward and reverse primers and 6-carboxy fluorescein (FAM)-labeled TaqMan (hydrolysis) probes specific for mouse genes (Supplementary Table S2) were designed to span an intron/exon junction where possible (Biosearch Technologies, Inc., Novato, CA, USA). All quantitative PCR reactions for each gene were performed on one plate, in 10 μL 96-well reactions containing SensiFAST™ Probe Lo-Rox (BioRad, West Ryde, NSW, Australia) using the ViiA™ 7 Real-Time PCR System (Life Technologies™, Mulgrave, VIC, Australia). Normalized data (ΔC_t_) were analysed using the 2^−∆∆*C*t^ method and presented as mean ± SEM as described above.

### 4.10. Reagents

All drugs were purchased from Sigma-Aldrich (St Louis, MO, USA), except for ODQ and U46619 (Cayman Chemical, Ann Arbor, MI, USA). All drugs were dissolved in deionized water with the exception of Indo, which was dissolved in 0.1 mol/L sodium carbonate, and U46619, which was dissolved in 100% ethanol. All subsequent dilutions were in deionized water. Cmpd17b and Cmpd43 were first dissolved in 100% DMSO as stock solution (10 mmol/L^−1^), subsequent dilution was in 50% DMSO/50% Krebs, and further dilutions were in Krebs.

### 4.11. Statistical Analysis

All results are expressed as a mean ± SEM and n representing the number of animals per group. Concentration–response curves were computer fitted to a sigmoidal curve using nonlinear regression (Prism version 7.0, GraphPad Software, San Diego, CA, USA) to calculate the sensitivity of each agonist (pEC_50_). Maximum relaxation (R_max_) was expressed as a percentage of the level of precontraction to U46619. Concentration–response curves were also analysed with repeated measures two-way ANOVA (treatment vs. concentration). Body weight, blood glucose, HbA_1c_, pEC50, and Rmax values were compared within the single variable of treatment using one-way ANOVA with post hoc analysis using the Tukey’s, Dunnett’s or independent t-tests where appropriate. A normality test was performed using D’Agostino-Pearson Omnibus and Shapiro Wilk normality test as appropriate (where sample size allow), and all data except for Figure 4A–C were normally distributed. If the data were not normally distributed, non-parametric analysis (Kruskal–Wallis test) was used for statistical analysis. A level of *P* < 0.05 was considered statistically significant. Outliers were excluded in data analysis and presentation, where indicated. An outlier was predefined when an individual data point was 2 standard deviations from the mean.

### 4.12. Chemical Compounds

Chemical compounds studied in this article ACh (PubChem CID: 6060); BOC2 (PubChem CID: 132822); CsH (PubChem CID: 6476564); DMSO (PubChem CID: 679); Indo (PubChem CID: 3715); L-NAME (PubChem CID: 39836); Nifedipine (PubChem CID: 4485); ODQ (PubChem CID: 1456); Quin-C7 (PubChem CID: 11647668); STZ (PubChem CID: 57654595); SNP (PubChem CID: 329763522); TRAM-34 (PubChem CID: 656734); U46619 (PubChem CID: 5311493).

## 5. Conclusions

In conclusion, the small-molecule FPR agonist, Cmpd17b is a direct vasodilator in normal healthy mouse aorta with actions on the vascular smooth muscle cells. More importantly, this vasodilator capacity of Cmpd17b is preserved in diabetes, which is typified by vascular dysfunction. This suggests that in an acute setting, Cmpd17b is an effective vasodilator, which may be used to reduce blood pressure clinically for diabetic patients, who are NO resistance [58]. Furthermore, chronic treatment with Cmpd17b *in vivo* for eight weeks reduced vascular inflammation, which is well-recognized as an important contributor to endothelial dysfunction. This may have, in part, resulted in the improvement of endothelial function in diabetic mice, suggesting that it could function as a vasoprotective drug in the context of this disease. This study facilitates new therapeutic opportunities to develop FPR agonists for the treatment of vascular diseases. Specifically, given that diabetic vasculopathy is more pronounced in resistance-like small vessels, the vascular effects of Compd17b in small vessels will be investigated in future studies.

## Figures and Tables

**Table 1:** Effects of various pharmacological inhibitors on vascular reactivity of Cmpd17b in the aorta.

**Agonist**	**Sample Size**	**Cmpd17b**	
	*n*	*pEC_50_*	*R_max_*
Control (DMSO)	12	5.17 ± 0.06	69 ± 5
L-NAME	8	5.08 ± 0.04	72 ± 2
Indo	4	5.09 ± 0.15	74 ± 4
Indo+L-NAME	4	5.17 ± 0.10	67 ± 8
Indo+L-NAME+KCaB	4	5.27 ± 0.06	75 ± 8
Endothelium intact	6	5.07 ± 0.03	62 ± 5
Endothelium denuded	6	5.06 ± 0.03	55 ± 10
Control (DMSO)	6	5.09 ± 0.05	60 ± 2
50mM K^+^	4	5.13 ± 0.05	73 ± 8
ODQ	5	5.14 ± 0.09	71 ± 2
Glibenclamide	6	5.20 ± 0.18	52 ± 3
**Agonist**		**CaCl_2_**	
	*n*	*pEC_50_*	*E_max_*
Control (DMSO)	3	2.34 ± 0.23	111 ± 10
Cmpd17b	3	ND	0 *
Nifedipine	3	ND	0 *

Values are expressed as mean ± S.E.M; *n*, aorta from individual mice. DMSO, vehicle control; R_max_, maximum relaxation; E_max_, maximum constriction relative to %KPSS; Indo, indomethacin; KCaB, combination of the small and intermediate conductance calcium-activated potassium channel inhibitors apamin and TRAM34; L-NAME, Nω-nitro-L-arginine methyl ester; ODQ, 1H-[1,2,4]oxadiazolo[4 ,3-a]quinoxalin-1-one; pEC_50_, sensitivity;. ND = not determined. * Significantly different from DMSO control (by one-way ANOVA, Dunnett’s post hoc test).

**Table 2:** Vascular reactivity of aorta isolated from of mice treated with citrate buffer (control), STZ (diabetic), or STZ and Cmpd17b (diabetic+Cmpd17b).

Drug	Sample Size	Control		Sample Size	Diabetic		Sample Size	Diabetic +Cmpd17b	
*ACh*	*n*	*pEC_50_*	*R_max_*	*n*	*pEC_50_*	*R_max_*	*n*	*pEC_50_*	*R_max_*
Control	6	7.09 ± 0.07	92 ± 1	9	6.70 ± 0.06 *	88 ± 2	7	7.11 ± 0.11 ^#^	90 ± 2
Indo	7	6.97 ± 0.12	91 ± 1	7	6.82 ± 0.12	88 ± 3	7	6.68 ± 0.11^	86 ± 2
Indo+L-NAME	7	ND	9 ± 4^	9	ND	13 ± 6^	8	ND	11 ± 8 ^
*SNP*	*n*	*pEC_50_*	*R_max_*	*n*	*pEC_50_*	*R_max_*	*n*	*pEC_50_*	*R_max_*
Control	7	7.55 ± 0.11	95 ± 1	7	7.80 ± 0.12	96 ± 1	7	7.58 ± 0.08	94 ± 1
*Cmpd17b*	*n*	*pEC_50_*	*R_max_*	*n*	*pEC_50_*	*R_max_*	*n*	*pEC_50_*	*R_max_*
Control	*6*	5.12 ± 0.13	70 ± 5	*4*	5.14 ± 0.13	67 ± 5	ND	ND	ND

Values are expressed as mean ± S.E.M; *n*, aorta from individual mice. ACh, acetylcholine; Indo, indomethacin; L-NAME, Nω-nitro-L-arginine methyl ester; SNP, sodium nitroprusside; pEC_50_, sensitivity; R_max_, maximum relaxation. ND = not determined. * Significantly different from control treated group; # significantly different from diabetic group; ^ R_max_ significantly different from control and Indo incubated aorta within the same treatment group.

## Supplementary Materials

Supplementary materials can be found at https://www.mdpi.com/1422-0067/21/4/1384/s1.

## Abbreviations

ACh	Acetylcholine
Akt	Protein kinase B
Apamin	Small conductance calcium-activated potassium channel inhibitor
ANX-A	Annexin A1, endogenous ligand for FPR
cAMP	Cyclic adenosine monophosphate
CB	Citrate buffer
Cmpd17b	Compound 17b, agonist of FPR1/2
Cmpd43	Compound 43, agonist of FPR1/2
COX 1	Cyclooxygenase-1
COX 2	Cyclooxygenase-2
Cq	Quantification cycle
DMSO	Dimethyl sulfoxide
EDH	Endothelium-derived hyperpolarization
Emax	Maximum contraction
ERK1/2	Extracellular signal-regulated kinases 1/2
FPR	Formyl peptide receptor family
FPR1	Formyl peptide receptor 1
FPR2	Formyl peptide receptor 2
Glibenclamide	ATP-sensitive potassium (KATP) channel blocker
HbA1c	Glycated haemoglobin A1c
IKca	Intermediate conductance calcium-activated potassium channel
KCaB	Combination of inhibitors (Apamin and TRAM34)
Indo	Indomethacin
L-NAME	nitric oxide synthase (NOS) inhibitor, L-NG-Nitroarginine methyl ester
LxA4	Lipoxin 4
NGS	Normal goat serum
Nifedipine	Calcium channel blocker
NO	Nitric oxide
NOS	Nitric oxide synthase
ODQ	Soluble guanylate cyclase inhibitor, 1H-(1,2,4)oxadiazolo[4,3-a]quinoxalin-1-one
PBS	Phosphate buffered saline
pEC50	Sensitivity
PGI2	Prostacyclin
PSS	Physiological saline solution
PTGIR	Prostaglandin I2 receptor
PTGIS	Prostacyclin synthase
Rn18s	18s Ribosomal RNA
Rmax	Maximum relaxation
SKca	Small conductance calcium-activated potassium channel
SNP	Sodium nitroprusside
STZ	Streptozotocin
TRAM34	Intermediate conductance calcium-activated potassium channel inhibitor, 1-[(2-Chlorophenyl)diphenylmethyl]-1H-pyrazole
U46619	Thromboxane A2 mimetic
