# Supplementary material for: The Novel Small-molecule Annexin-A1 Mimetic, Compound 17b, Elicits Vasoprotective Actions in Streptozotocin-induced Diabetic Mice
Source: Int J Mol Sci. 2020 Feb 18;21(4):1384. doi: 10.3390/ijms21041384 (PMC7073122; doi:10.3390/ijms21041384)
Supplement: Supplementary file 1 [file ijms-21-01384-s001.pdf]

Supplementary Materials:

The Novel Small-molecule Annexin-A1 Mimetic, Compound 17b, Elicits Vasoprotective Actions in Streptozotocin-induced Diabetic Mice

Sarah A Marshall [1,†], Cheng Xue Qin [2,3,4,†,*], Maria Jelinic [5], Kelly O'Sullivan [6], Minh Deo [2], Jesse Walsh [2], Mandy Li [2], Laura J Parry [6], Rebecca H. Ritchie [2,3,4,‡] and Chen Huei Leo [6,7,‡,*]

1 The Ritchie Centre, Department of Obstetrics and Gynaecology, School of Clinical Sciences, Monash University, Clayton, Victoria 3168 Australia; sarah.marshall@monash.edu

2 Heart Failure Pharmacology, Baker Heart & Diabetes Institute, Victoria 3004, Australia; Minh.Deo@baker.edu.au (M.D.); jesselorenwalsh15@gmail.com (J.W.); Mandy.Li@baker.edu.au (M.L.); Rebecca.Ritchie@monash.edu (R.H.R.)

3 Department of Pharmacology, University of Melbourne, Victoria 3010, Australia

4 Drug Discovery Biology, Monash Institute of Pharmaceutical Sciences, Monash University, Victoria 3052, Australia

5 Department of Physiology, Anatomy & Microbiology, La Trobe University, Bundoora, Victoria 3086, Australia; M.Jelinic@latrobe.edu.au

6 School of BioSciences, The University of Melbourne, Victoria 3010, Australia; kelly.osullivan@monash.edu (K.O.S.); laura.parry@adelaide.edu.au (L.J.P.)

7 Science and Math Cluster, Singapore University of Technology & Design, Singapore 487372, Singapore

* Correspondence: chenhuei_leo@sutd.edu.sg (C.H.L.); ChengxueHelena.Qin@baker.edu.au (C.X.Q.)

† These authors contributed equally to this work.

‡ Joint senior authors.

Supplementary Figure

A

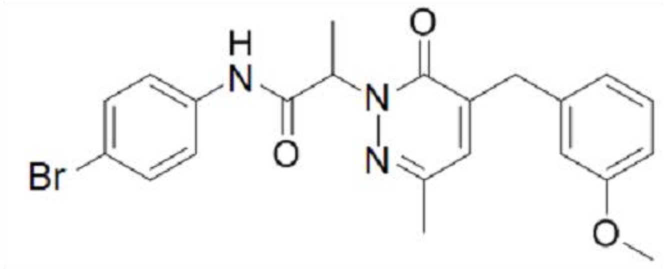

Compound 17b (Cmpd17b)

B

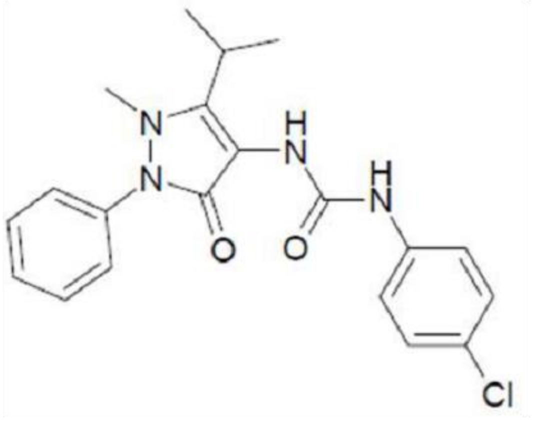

Compound 43 (Cmpd43)

Supplementary Figure S1: Crystalline structure of (**A**) Cmpd17b and (**B**) Cmpd43.

Supplementary Table S1: Primer sequences for the quantitative amplification of murine genes using SYBR green.

Gene	Accession Number	Oligo Type	Primer Sequence 5'–3'	Amplicon Length
Rn18S	NR_003278	Forward	TGTTCACCATGAGGCTGAGATC	67 bp
		Reverse	TGGTTGCCTGGGAAAATCC	
Fpr1	NM_013521	Forward	CCTTGGCTTTCTTCAACAGC	247 bp
		Reverse	GCACAGTGGAACTCAAAGCA	
Fpr2	NM_008039	Forward	ACAGCAGTTGTGGCTTCCTT	192 bp
		Reverse	CCTGGCCCATGAAAACATAG	
Fpr3	NM_008042	Forward	GGGCAACTCTGTTGAGGAAA	201 bp
		Reverse	GAAGGAAGCCACAACTCCTG	

Supplementary Table S2: Primer and probe sequences for the quantitative amplification of murine genes using TaqMan.

Gene	Accession Number	Oligo Type	Primer Sequence 5'–3'	Amplicon Length
18S	NR_003278	Forward	GCATGGCCGTTCTTAGTTGG	67 bp
		Reverse	TGCCAGAGTCTCGTTCGTTA	
		Probe	TGGAGCGATTTGTCTGGTTATTCCGA	
Fpr1	NM_013521	Forward	TCTTCCTGATTGCCCTCATT	121 bp
		Reverse	GCACAAATCCAGGGTACGAT	
		Probe	GTGAGCCTAGCCAAGAAGGTAA	
Fpr2	NM_008039	Forward	TGATTGCTCTCATTGCCTTG	62 bp
		Reverse	GTTCTGAGCCCAGACTGGAT	
		Probe	GACCGCTGCATTTGTGTTCT	
Ptgs1	NM_008969	Forward	TCGGTCCTGCTCGCAGAT	95 bp
		Reverse	AGGCCAAAGCGGACACAGA	
		Probe	CACCAGTCAATCCCTGTTGTTA	
Ptgs2	NM_011198	Forward	TCCTCCCGTAGCAGATGAC	158 bp
		Reverse	TGCTGGGCAAAGAATGCAAAC	
		Probe	GGGAAATAAGGAGCTTCCTGAT	
Ptgis	NM_008968	Forward	GGGGCAGATACGTCACTGTT	78 bp

		Reverse	GAAGATGGCATAGGGATGGA	
		Probe	TTACGACACAGTGGTGTGGG	
Ptgir	NM_008967	Forward	GCTCGTTTGTACCGACCTCT	121 bp
		Reverse	TGAGTGAAGCCTCGGATCAT	
		Probe	GGCACGAGAGGATGAAGTTT	
Tnf	NM_001278601	Forward	GGCTGCCCCGACTACGT	66 bp
		Reverse	TTTCTCCTGGTATGAGATAGCAAATC	
		Probe	TCACCCACACCGTCAG	
Mcp-1 (Ccp1)	NM_011333	Forward	GTCTGTGCTGACCCCAAGAAG	62 bp
		Reverse	TGGTTCCGATCCAGGTTTTTA	
		Probe	AATGGGTCCAGACATAC	
Icam-1	NM_010493	Forward	GGAGGTGGCGGGAAAGTT	72 bp
		Reverse	TCCAGCCGAGGACCATACAG	
		Probe	CTGAAAGCTCTCCACCTC	
IL1β	NM_008361	Forward	TCGTGCTGTCGGACCCATA	93 bp
		Reverse	CTTGTACAAAGCTCATGGAGAATATCAC	
		Probe	CTGAAAGCTCTCCACCTC	

Supplementary Table S3: Vascular reactivity of small molecule FPR agonists, Cmpd17 and Cmpd43 in the mouse. Values are expressed as mean ± S.E.M; *n*, aorta from individual mice. ACh, acetylcholine; Cmpd17b, compound 17b; Cmpd43, compound 43; DMSO, placebo control; E_{max}, maximum constriction relative to %U46619; pEC_{50}, sensitivity; R_{max}, maximum relaxation (negative values represent the reversal of relaxation). ND = not determined. *Significantly different from DMSO control (by one-way ANOVA, Tukeys *post hoc* test).

	**DMSO**			**Cmpd17b**			**Cmpd43**		
	n	pEC_{50}	R_{max}	*n*	pEC_{50}	R_{max}	*n*	pEC_{50}	R_{max}
Direct vasodilation (FPR agonists)	5	ND	-6 ±7	5	5.09 ±0.03	62 ±5*	5	ND	-2 ±9
ACh	5	6.82 ±0.15	93 ±2	5	6.95 ±0.19	96 ±1	5	7.04 ±0.09	93 ±3
	n	pEC_{50}	E_{max}	*n*	pEC_{50}	E_{max}	*n*	pEC_{50}	E_{max}
U46619	5	7.65 ±0.06	112 ±1	5	7.22 ±0.06*	96 ±4*	5	7.51 ±0.11	108 ±3
